# Reduced beta cell number rather than size is a major contributor to beta cell loss in type 2 diabetes

**DOI:** 10.1007/s00125-021-05467-7

**Published:** 2021-05-03

**Authors:** Hironobu Sasaki, Yoshifumi Saisho, Jun Inaishi, Yuusuke Watanabe, Tami Tsuchiya, Masayoshi Makio, Midori Sato, Masaru Nishikawa, Minoru Kitago, Taketo Yamada, Hiroshi Itoh

**Affiliations:** 1 Department of Internal Medicine, Keio University School of Medicine, Tokyo, Japan; 2 Center for Preventative Medicine, Keio University School of Medicine, Tokyo, Japan; 3 Department of Surgery, Keio University School of Medicine, Tokyo, Japan; 4 Department of Pathology, Keio University School of Medicine, Tokyo, Japan; 5 Department of Pathology, Saitama Medical University, Saitama, Japan

**Keywords:** Beta cell mass, Beta cell number, Beta cell size, Human pancreas, Japanese

## Abstract

**Aims/hypothesis:**

Type 2 diabetes is characterised by reduced beta cell mass (BCM). However, it remains uncertain whether the reduction in BCM in type 2 diabetes is due to a decrease in size or number of beta cells. Our aim was to examine the impact of beta cell size and number on islet morphology in humans with and without type 2 diabetes.

**Methods:**

Pancreas samples were obtained from 64 Japanese adults with (*n* = 26) and without (*n* = 38) type 2 diabetes who underwent pancreatectomy. Using pancreatic tissues stained for insulin, we estimated beta cell size based on beta cell diameter. Beta cell number was estimated from the product of fractional beta cell area and pancreas volume divided by beta cell size. The associations of beta cell size and number with islet morphology and metabolic status were examined.

**Results:**

Both beta cell size (548.7 ± 58.5 vs 606.7 ± 65.0 μm^3^, *p* < 0.01) and number (5.10 × 10^8^ ± 2.35 × 10^8^ vs 8.16 × 10^8^ ± 4.27 × 10^8^, *p* < 0.01) were decreased in participants with type 2 diabetes compared with those without diabetes, with the relative reduction in beta cell number (37%) being greater than for beta cell size (10%). Beta cell number but not size was positively correlated with BCM in participants with and without type 2 diabetes (*r* = 0.97 and *r* = 0.98, both *p* < 0.01) and negatively correlated with HbA_1c_ (*r* = −0.45, *p* < 0.01).

**Conclusions/interpretation:**

Both beta cell size and number were reduced in participants with type 2 diabetes, with the relative reduction in beta cell number being greater. Decrease in beta cell number appears to be a major contributor to reduced BCM in type 2 diabetes.

**Graphical abstract:**

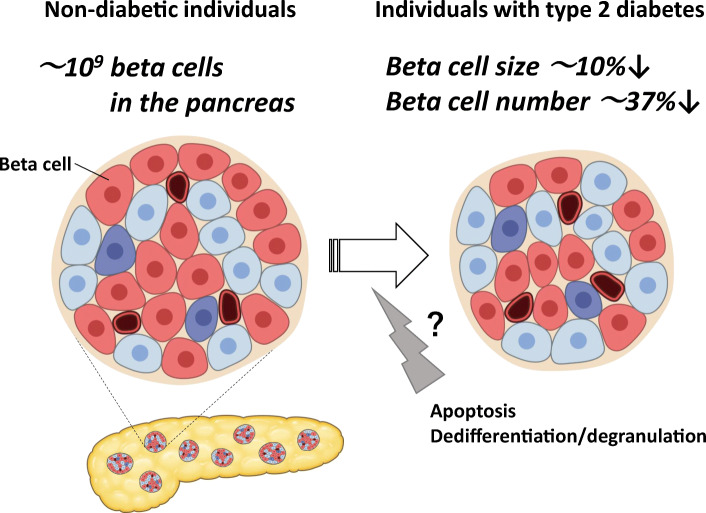

**Supplementary Information:**

The online version of this article (10.1007/s00125-021-05467-7) contains peer-reviewed but unedited supplementary material.

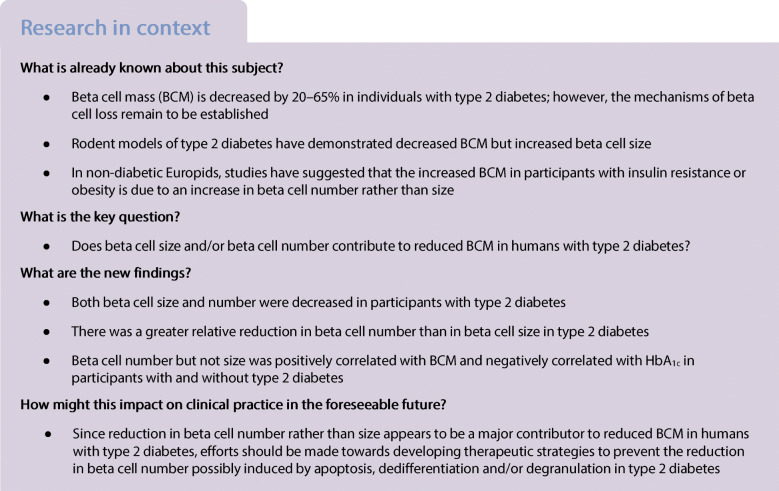



## Introduction

Type 2 diabetes is characterised by reduced beta cell mass (BCM) [1]. Since type 2 diabetes is a progressive disorder, it is important to develop treatment strategies to preserve BCM in individuals with type 2 diabetes [2].

Although BCM has been shown to decrease by approximately 20–65% in individuals with type 2 diabetes [1, 3, 4], the precise mechanism remains uncertain. Increased beta cell apoptosis in individuals with type 2 diabetes has been reported as one of the underlying mechanisms of reduced BCM [3]; however, recent studies have also suggested beta cell dedifferentiation and/or degranulation as possible mechanisms [5, 6]. Rodent models of type 2 diabetes have shown decreased BCM but increased beta cell size [7]. In a previous study on Europids, beta cell number rather than size was increased in non-diabetic individuals with insulin resistance or obesity [8, 9]. However, because the studies did not compare these findings with those in diabetic individuals, it remains unclear how beta cell size and number are altered by diabetes. Therefore, using our previously published data [10], we here aimed to estimate the relative contribution of beta cell size and number to the reduction of BCM in individuals with type 2 diabetes.

## Methods

### Participants

The characteristics of the participants have been reported previously [10] and are shown in ESM Table 1. The Ethics Committee of Keio University School of Medicine approved this study. Further information can be found in ESM Methods. Briefly, 64 Japanese individuals with (*n* = 26) and without (*n* = 38) diabetes were included in this study.

### Measurements and questionnaire

Information about pancreatic disease, surgical procedure, and height and weight at the time of surgery was obtained from the medical records. Preoperative HbA_1c_ was measured by HPLC (HLC723G11; Tosoh, Tokyo, Japan). Participants were asked about their detailed weight trajectory using a questionnaire, as previously reported [10].

### Pancreatic tissue processing

Surgically removed pancreatic specimens were quickly fixed in formaldehyde and embedded in paraffin for subsequent analysis. Then, 5 μm sections were cut from the tumour-free area and stained for light microscopy as follows: (1) with haematoxylin–eosin; (2) for insulin (peroxidase staining) with haematoxylin; (3) for glucagon with haematoxylin; and (4) for insulin and Ki67 for assessment of beta cell replication, as previously described [10–13].

### Morphological analysis

To analyse the pancreatic tissues, a single cross-sectional pancreatic section for each participant was used. The entire pancreatic section containing approximately 300 islets (total pancreas area 126 ± 50 mm^2^) was imaged at the original magnification of ×200 (×20 objective) using a NanoZoomer-XR slide scanner and viewed with NDP.view2 software (Hamamatsu Photonics, Shizuoka, Japan).

For analysis of islet morphology, the ratio of BCA to total pancreas area was digitally measured using Image Pro Premier software (Media Cybernetics, Silver Spring, MD, USA). All analyses were conducted by a single researcher (H. Sasaki), and inter- and intra-observer coefficient of variance were approximately 11% and 5%, respectively. Individual pancreatic tissues were analysed twice, with blinding to the metabolic status such as BMI and HbA_1c_, and the average of the two measurements was used, as previously described [10].

For further morphological analysis, islet density and mean islet size were quantified using NDP.view2 in randomly selected areas of pancreatic tissue containing at least 100 islets in each case (105 ± 5 islets, total 6741 islets) [10, 12, 13]. In addition, we quantified scattered beta cells, insulin-positive duct cells and beta cell replication (i.e. double staining for insulin and Ki67) as surrogate markers for beta cell turnover. The frequency of beta cell apoptosis was not assessed in this study because it was extremely rare, as described in previous reports [11–13].

Scattered beta cells were defined as a cell cluster of no more than three beta cells in acinar tissue, and the density of scattered beta cells was defined as the number of scattered beta cells/pancreas area (no./mm^2^). The density of insulin-positive duct cells was also measured and expressed as the number of insulin-positive duct cells/pancreas area (no./mm^2^). Beta cell replication frequency was expressed as the percentage of islets with Ki67.

To measure the size of individual beta cells, six islets were randomly selected from each sample using NDP.view2 software, as previously reported [11]. These islets were then examined to identify six representative beta cells within each. We primarily selected cells with a circular shape and that were judged by the observer to have been sectioned through their maximum diameters. To determine the mean beta cell diameter, six distances between two adjacent cell nuclei (including one of the nuclei) were measured in each of the six islets (i.e. a total of 36 diameters in each case).

### Estimation of beta cell size and number

To estimate beta cell size, mean beta cell diameter was used. As the cells are not entirely circular, we estimated beta cell size as the average of two values calculated as 4πr^3^/3 (sphere) and 8r^3^ (cube) (where r = half the mean beta cell diameter).

Beta cell number was calculated, using the reference values of pancreas volume [14], by the following formula:

$$ \mathrm{Beta}\ \mathrm{cell}\ \mathrm{number}=\mathrm{Parenchymal}\ \mathrm{pancreas}\ \mathrm{volume}\ \left({\upmu \mathrm{m}}^3\right)\times \mathrm{BCA}\div \mathrm{Beta}\ \mathrm{cell}\ \mathrm{size}\ \left({\upmu \mathrm{m}}^3\right) $$

The obtained pancreas volume was multiplied by 0.92 in the diabetes group because pancreas parenchymal volume was reduced by 8% in participants with type 2 diabetes [14] and BCM was estimated as the product of BCA (%) and pancreas weight (g), assuming 1 cm^3^ pancreas = 1 g.

### Statistical analysis

Data are presented as mean ± SD unless otherwise specified. Mann–Whitney *U* test was used to analyse the differences between the two groups, and Spearman correlation coefficients were used to examine the correlation between two variables. A *p* value <0.05 was taken to indicate statistical significance. All analyses were performed using SPSS (version 26; SPSS, IBM, Chicago, IL, USA).

## Results

### Participant characteristics

The characteristics of the 38 non-diabetic participants and 26 diabetic participants have been reported previously [10] and are shown in ESM Table 1. As reported, BCA and estimated BCM were reduced by 34% and 43%, respectively, in the participants with diabetes (DM group) when compared with participants without diabetes (NDM group) (ESM Table 1). Representative photographs of islets from participants in the NDM and DM groups are shown in ESM Fig. 1.

### Effects of diabetes and obesity on beta cell size and number

**Fig. 1:**
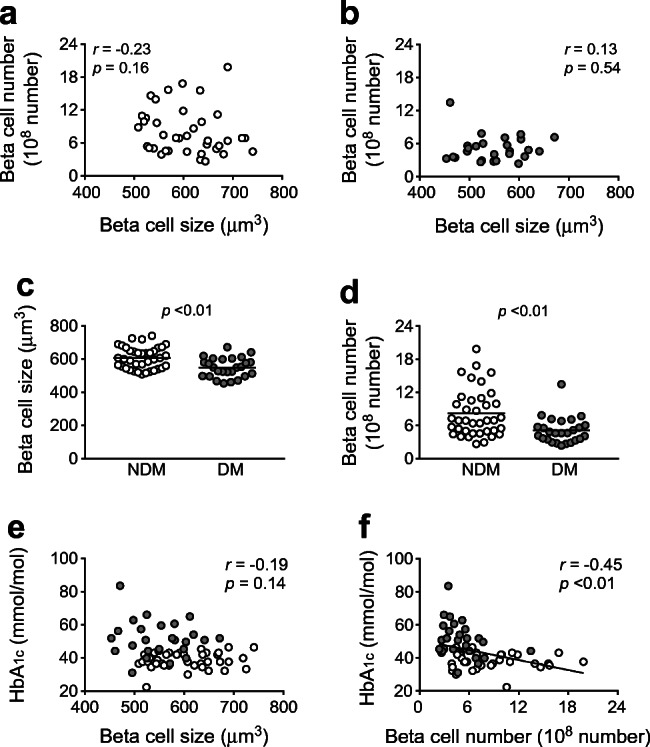
(**a**, **b**) Correlation between beta cell size and number in the NDM group (**a**) and the DM group (**b**). (**c**, **d**) Effects of diabetes on beta cell size (**c**) and number (**d**). (**e**, **f**) Correlation between HbA_1c_ and beta cell size (**e**) and number (**f**). Grey circles, DM group; white circles, NDM group. Bars indicate mean

Overall, mean beta cell diameter was 9.13 ± 0.36 μm in the total participants. As a result, beta cell size and number were calculated as 583.1 ± 68.3 μm^3^ and 6.92 × 10^8^ ± 3.90 × 10^8^, respectively, indicating a wider inter-individual variation in beta cell number than in beta cell size (ESM Table 1). There was no significant correlation between beta cell size and number in either the NDM group (*r* = −0.23, *p* = 0.16; Fig. 1a) or the DM group (*r* = 0.13, *p* = 0.54; Fig. 1b).

Both beta cell size (548.7 ± 58.5 vs 606.7 ± 65.0 μm^3^, *p* < 0.01; Fig. 1c and ESM Table 1) and number (5.10 × 10^8^ ± 2.35 × 10^8^ vs 8.16 × 10^8^ ± 4.27× 10^8^, *p* < 0.01; Fig. 1d and ESM Table 1) were significantly reduced in the DM group compared with the NDM group. However, in the DM group, the relative reduction was greater for beta cell number (37%) than for beta cell size (10%). In both groups of participants overall, HbA_1c_ was not correlated with beta cell size (*r* = −0.19, *p* = 0.14; Fig. 1e) but was negatively correlated with beta cell number (*r* = −0.45, *p* < 0.01; Fig. 1f) as well as with BCA (*r* = −0.38, *p* < 0.01) and BCM (*r* = −0.47, *p* < 0.01).

In this Japanese cohort, there was no correlation between current BMI (ESM Fig. 2) or maximum BMI (data not shown) and beta cell size or number in either the NDM group or the DM group.

### Effects of beta cell size and number on islet morphological characteristics

**Fig. 2:**
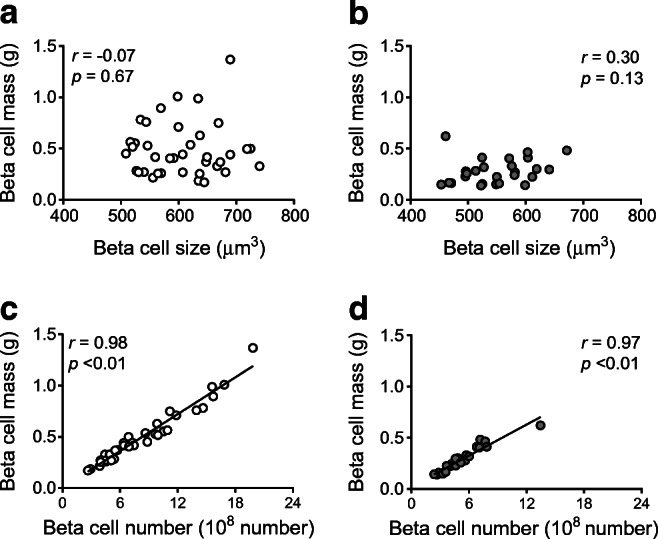
Correlation between beta cell size and estimated BCM in the NDM group (**a**) and the DM group (**b**) and between beta cell number and estimated BCM in the NDM group (**c**) and the DM group (**d**). Grey circles, DM group; white circles NDM group

There was no correlation between beta cell size and islet morphology in either the NDM group or the DM group (Fig. 2a,b and ESM Figs. 3, 4). On the other hand, there was a strong correlation between beta cell number and BCM in both the NDM and DM groups (*r* = 0.98 and 0.97, respectively, both *p* < 0.01; Fig. 2c,d), indicating that BCM was mostly determined by beta cell number rather than size. There was also a positive correlation between beta cell number and islet density, and number of scattered beta cells in the NDM and DM groups (ESM Figs. 3c,d, 4c,d). Regarding other markers of beta cell turnover, there was no correlation between beta cell number and number of insulin-positive duct cells or beta cell replication in either the NDM group or the DM group (ESM Fig. 4g,h,k,l).

## Discussion

In the present study, we found that both beta cell size and beta cell number were decreased in participants with type 2 diabetes. However, because the reduction in beta cell number was greater than the reduction in beta cell size (37% vs 10%), beta cell number is likely to have a major role with respect to the reduced BCM in type 2 diabetes. We also found that beta cell number, as well as BCA and BCM, but not beta cell size was negatively correlated with HbA_1c_, indicating the importance of beta cell number for glycaemic control. Furthermore, in this study, beta cell number was strongly correlated with BCM and islet density. We reported previously that islet density is strongly correlated with BCA and is a major determinant of BCM [15], consistent with the results of the present study, suggesting that beta cell number is a determinant of BCM through islet number. In this present cohort, we reported a significant positive correlation between birthweight and BCM, with no change in beta cell size, indicating an association between reduced beta cell number and low birthweight [10]. With regard to beta cell turnover, previous studies have suggested that beta cell neogenesis, rather than beta cell replication, is important as the mechanism of regulation of BCM in adults [3, 8, 9]. These results are consistent with the results of the present study, in which the number of scattered beta cells, but not replication of beta cells, was correlated with beta cell number. The close correlations among beta cell number, number of scattered beta cells, islet density and BCM could suggest an important role for newly formed beta cells (through neogenesis) in the regulation and maintenance of BCM in adult humans.

In this study, we found no correlation between beta cell size or number and BMI, in contrast to the previous study in Europids that showed an increase in beta cell number in non-diabetic individuals with insulin resistance [8]. This might be due to the lower BMI of our cohort compared with that in the study in Europids, although we did not evaluate insulin sensitivity. However, it is worth noting that beta cell size was consistent in obese participants regardless of race [9], supporting the concept that BCM is regulated primarily by beta cell number rather than beta cell size in humans.

This study was subject to certain limitations. First, actual BCM, expressed as the product of pancreatic weight and BCA, was not determined because the presence of pancreatic disease made it difficult to measure pancreatic weight or volume. However, instead we simulated BCM using reference values of pancreas volume taking into account age, BMI and presence of type 2 diabetes [14]. Although estimated beta cell size based on mean beta cell diameter might be inaccurate, the relative difference in beta cell size and number should not be largely affected. Indeed, the results were not changed when we assessed beta cell size and number using only cell diameter and BCA without pancreas volume (data not shown). Second, different sites of pancreatic tissue were analysed in each individual according to the operation; however, the proportion of endocrine cells has been shown to be relatively consistent regardless of pancreatic site, except for the ventral portion of the pancreatic head [4]. Moreover, BCA is widely used as a surrogate marker for BCM; this is supported by the significant correlation between BCA and HbA_1c_ in this and prior studies [12, 13]. Third, the surgical procedures and comorbidities of the participants might have affected islet morphology; however, BCA in this cohort was not different from that in other studies (i.e. BCA 1~2% and BCM 0.6~1.2 g [16]). Fourth, as we used the average value of beta cell diameters, which were assessed in a small population of beta cells (1386 and 936 cells in the NDM group and DM group, respectively), we might overlook the heterogeneity of beta cell size.

In conclusion, both beta cell size and number were decreased in type 2 diabetes. The relative reduction in type 2 diabetes was greater for beta cell number than for beta cell size. Beta cell number but not size was positively correlated with BCM and negatively correlated with HbA_1c_. These findings indicate that beta cell number rather than size is a major contributor to reduced BCM in humans with type 2 diabetes.

## Supplementary Information

ESM (PDF 545 kb)

## Data Availability

The datasets generated during and/or analysed during the current study are available from the corresponding author on reasonable request.

## Abbreviations

BCA: Beta cell area
BCM: Beta cell mass
DM group: Participants with diabetes
NDM group: Participants without diabetes
