# Supplementary material for: Reduced beta cell number rather than size is a major contributor to beta cell loss in type 2 diabetes
Source: Diabetologia. 2021 May 3;64(8):1816–21. doi: 10.1007/s00125-021-05467-7 (PMC8245378; doi:10.1007/s00125-021-05467-7)
Supplement: Supplementary file 1 — (PDF 545 kb) [file 125_2021_5467_MOESM1_ESM.pdf]

Electronic supplementary material (ESM)

ESM Methods

Participants

At Keio University Hospital, 401 patients underwent pancreatectomy from May 2012 to March 2019. Among them, 64 Japanese patients with and without diabetes were included in this study. The inclusion criteria were: (1) gave written informed consent; (2) able to recall their weight trajectory; and (3) sufficient normal pancreatic tissue for histological analysis. The exclusion criteria were: (1) a functional neuroendocrine tumour such as glucagonoma or insulinoma; or (2) type 1 diabetes. All patients with diabetes had a diagnosis of type 2 diabetes before being diagnosed with pancreatic disease (mean duration of diabetes 8.8 ± 5.9 years, ESM Table 1). This study used the same cohort as our previous study [10], and some participants included in the present study (*n*=11) were also included in our previous study [12].

ESM Table 1 Participant characteristics

Characteristic	NDM group	DM group	Total
N (male/female)	38 (20/18)	26 (23/3)	64 (43/21)
Age, years	61.7 ± 14.3	67.2 ± 11.2	63.9 ± 13.3
Current BMI, kg/m^2	22.3 ± 3.6	25.1 ± 3.5**	23.4 ± 3.8
Maximum BMI, kg/m^2	24.9 ± 4.0	28.5 ± 3.8**	26.3 ± 4.3
HbA_{1c}, mmol/mol	38 ± 4	51 ± 10**	43 ± 10
HbA_{1c}, %	5.7 ± 0.5	6.9 ± 1.0**	6.2 ± 0.9
Plasma glucose, mmol/l [a]	6.0 ± 0.8	7.8 ± 2.3**	6.7 ± 1.8
Clinical diagnosis, *n* (%)			
Pancreatic cancer	13 (34)	14 (54)	27 (42)
Intraductal papillary mucinous neoplasm	8 (21)	4 (15)	12 (19)
Non-functional neuroendocrine tumour	9 (24)	2 (8)	11 (17)
Bile duct cancer	3 (8)	2 (8)	5 (8)
Duodenal papilla cancer	1 (3)	1 (4)	2 (3)
Other [b]	4 (11)	3 (12)	7 (11)
Operative procedure, *n* (%)			
Pancreatoduodenectomy	24 (63)	16 (62)	40 (63)
Distal pancreatectomy	12 (32)	8 (31)	20 (31)
Total pancreatectomy	2 (5)	2 (8)	4 (6)
Duration of diabetes, years	–	8.8 ± 5.9	–
Family history of diabetes in second-degree relative, *n* (%)	11 (29)	13 (50)	24 (38)
Pancreas histology			
BCA, %	1.14 ± 0.58	0.75 ± 0.34**	0.98 ± 0.53
BCM, g	0.49 ± 0.26	0.28 ± 0.12**	0.41 ± 0.24
Mean beta cell diameter, μm	9.26 ± 0.33	8.95 ± 0.32**	9.13 ± 0.36
Beta cell size, μm^3	606.70 ± 64.95	548.67 ± 58.53**	583.13 ± 68.28
Beta cell number	$8.16 \times 10^8 \pm 4.27 \times 10^8$	$5.10 \times 10^8 \pm 2.35 \times 10^8$**	$6.92 \times 10^8 \pm 3.90 \times 10^8$

Data are mean ± SD or n (%)

[a]Timing of blood sampling (i.e. fasting or postprandial) was not determined.

[b]Tumour-forming pancreatitis, disseminated sarcoma originating from small intestine, metastatic pancreatic tumour, solid pseudopapillary neoplasm, serous cystic neoplasm, gastrointestinal stromal tumour, and gastric cancer; *n*=1 for each.

**$p<0.01$ vs NDM

These data were previously reported in our prior study [10].

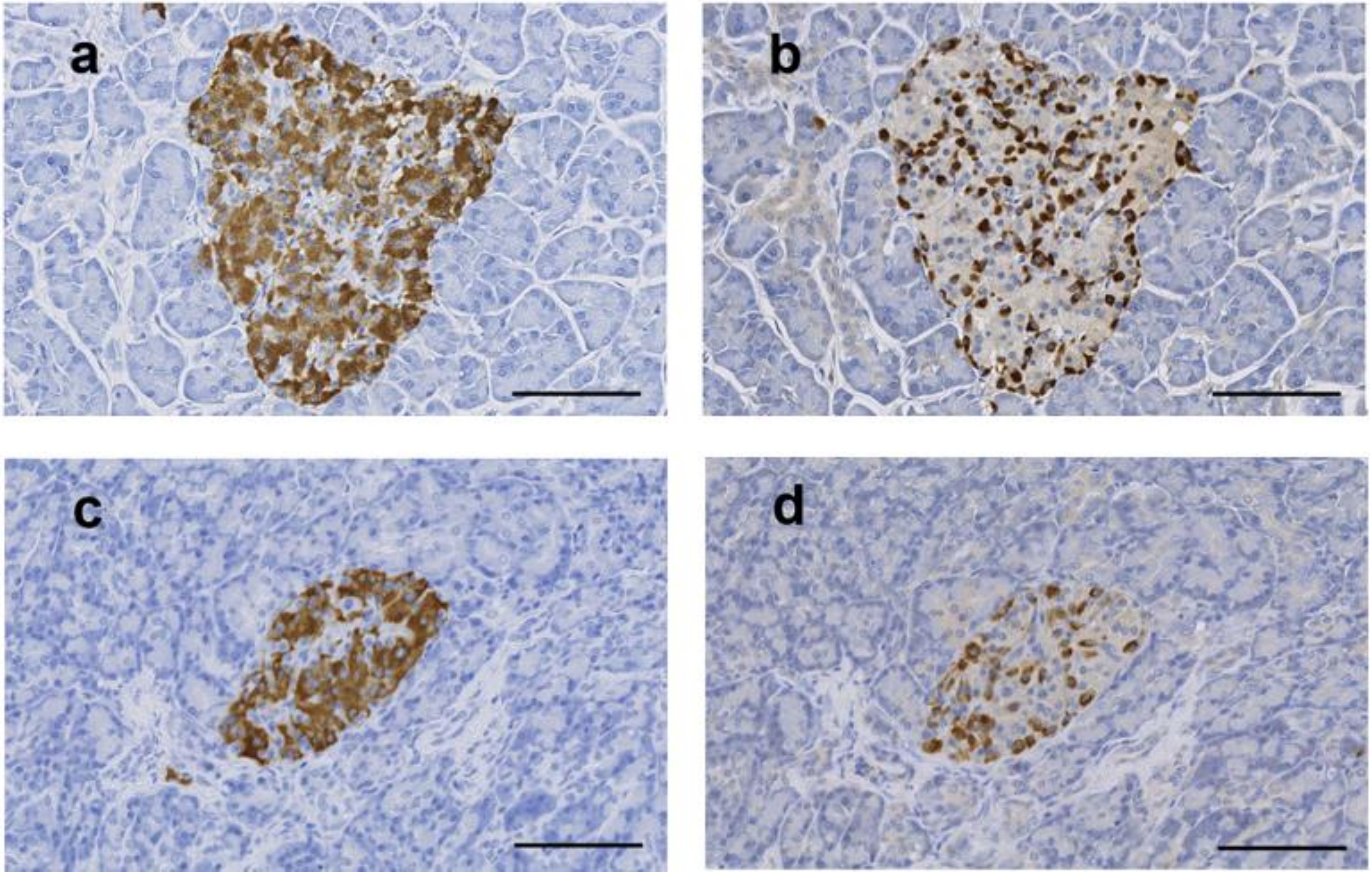
a
b
c
d

ESM Fig. 1 Representative photographs of islets immunostained for insulin and glucagon (brown). Examples of an islet stained for insulin (a) and glucagon (b) in a non-diabetic participant. Examples of an islet stained for insulin (c) and glucagon (d) in a diabetic participant. Scale bar, 100 μm.

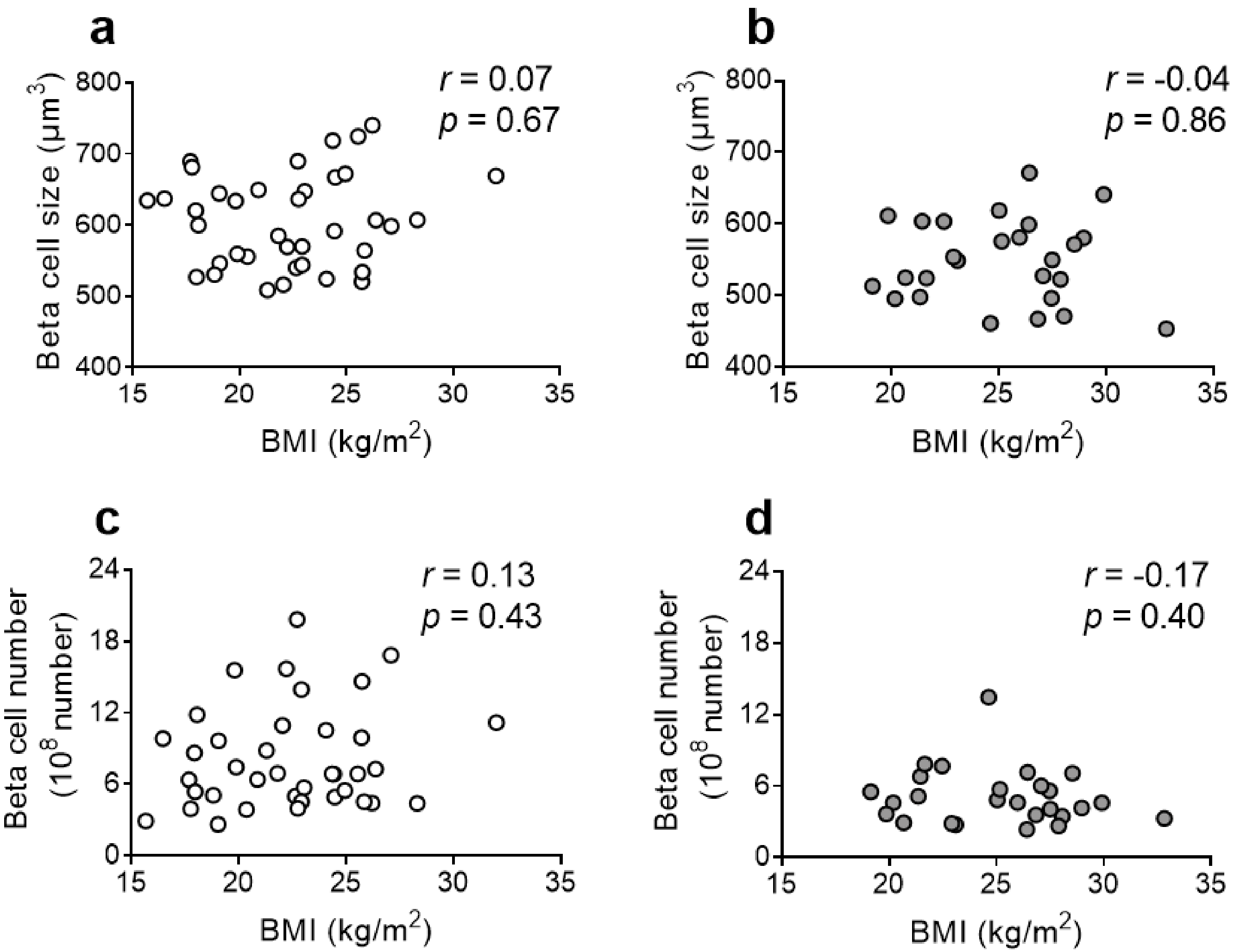
a
r = 0.07
p = 0.67
Beta cell size (μm3)
BMI (kg/m2)
b
r = -0.04
p = 0.86
Beta cell size (μm3)
BMI (kg/m2)
c
r = 0.13
p = 0.43
Beta cell number (10^8 number)
BMI (kg/m2)
d
r = -0.17
p = 0.40
Beta cell number (10^8 number)
BMI (kg/m2)

ESM Fig. 2 Correlation between beta cell size and current BMI in participants with (DM group) and without (NDM group) diabetes (a-b). Correlation between beta cell number and current BMI in participants with (DM group) and without (NDM group) diabetes (c-d). Grey and white circles show DM and NDM participants, respectively.

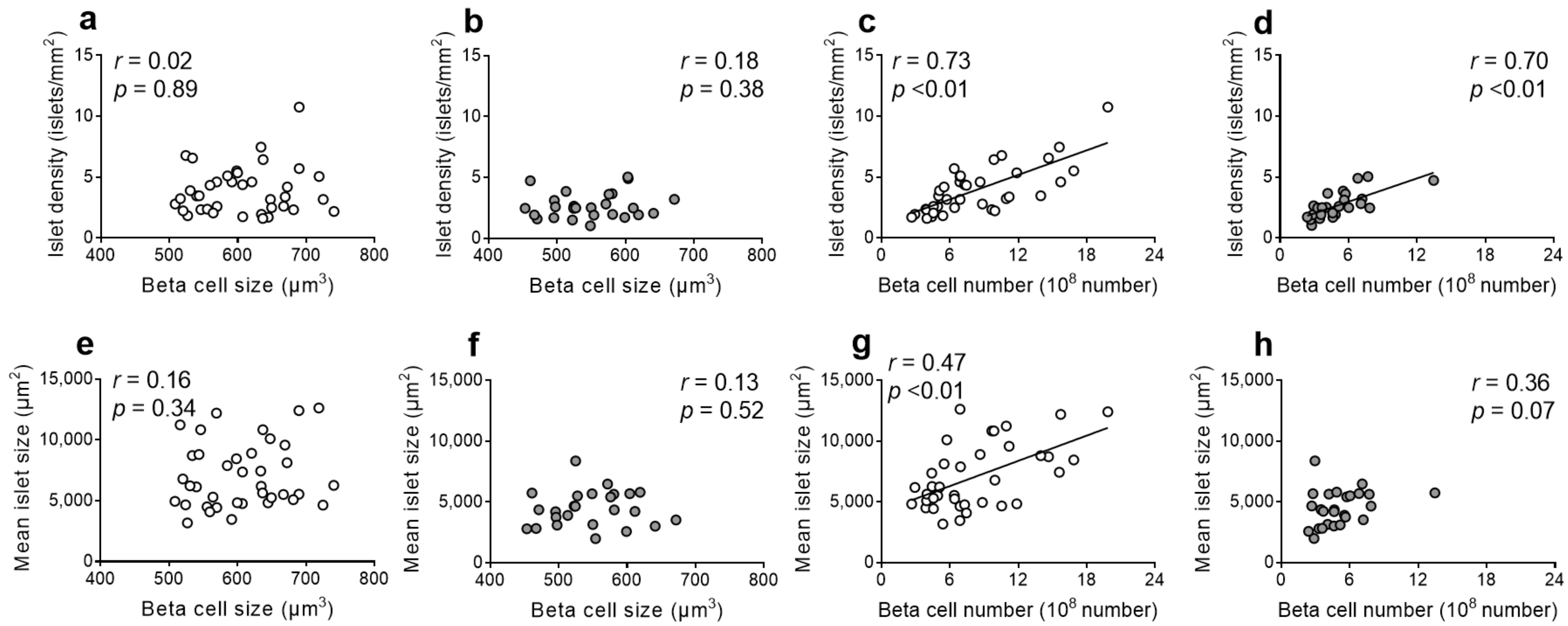

ESM Fig. 3 Correlation between beta cell size and islet density (a-b), and mean islet size (e-f) in participants with (DM group) and without (NDM group) diabetes. Correlation between beta cell number and islet density (c-d), and mean islet size (g-h) in participants with (DM group) and without (NDM group) diabetes. Grey and white circles show DM and NDM participants, respectively.

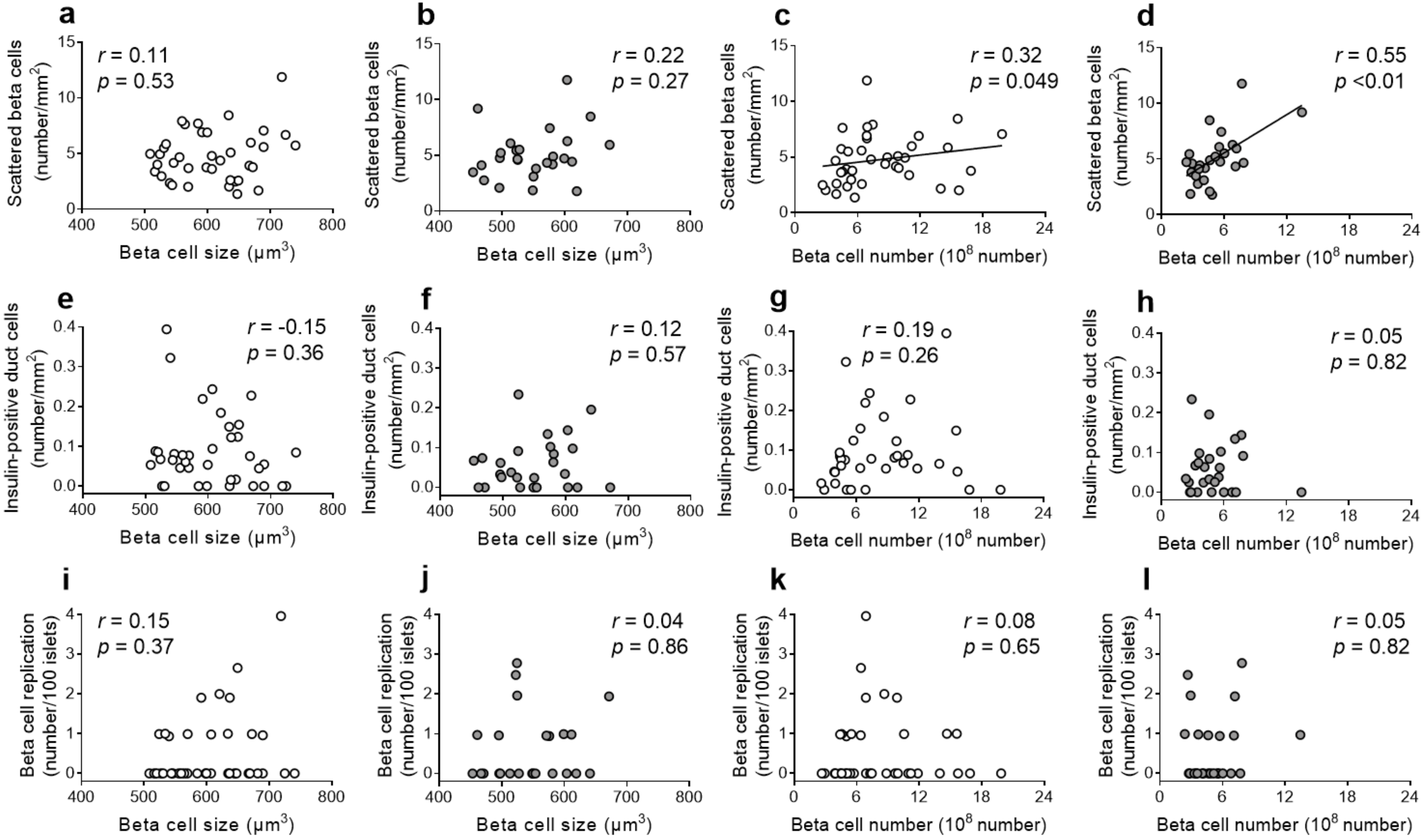
a
b
c
d
e
f
g
h
i
j
k
l
r = 0.11
p = 0.53
r = 0.22
p = 0.27
r = 0.32
p = 0.049
r = 0.55
p <0.01
r = -0.15
p = 0.36
r = 0.12
p = 0.57
r = 0.19
p = 0.26
r = 0.05
p = 0.82
r = 0.15
p = 0.37
r = 0.04
p = 0.86
r = 0.08
p = 0.65
r = 0.05
p = 0.82
Scattered beta cells (number/mm2)
Insulin-positive duct cells (number/mm2)
Beta cell replication (number/100 islets)
Beta cell size (µm3)
Beta cell number (108 number)

ESM Fig. 4 Correlation between beta cell size and number of scattered beta cells (a-b), number of insulin-positive duct cells (e-f), and beta cell replication (i-j) in participants with (DM group) and without (NDM group) diabetes. Correlation between beta cell number and number of scattered beta cells (c-d), number of insulin-positive duct cells (g-h), and beta cell replication (k-l) in participants with (DM group) and without (NDM group) diabetes. Grey and white circles show DM and NDM participants, respectively.
